# Nurses’ experience regarding patient safety in mobile pre-hospital care

**DOI:** 10.1590/0034-7167-2023-0529

**Published:** 2024-12-13

**Authors:** Eduardo Dias Filipe, Roberto Chrispim Modesto, Hercules de Oliveira Carmo, Haviley Oliveira Martins, Maristela Santini Martins

**Affiliations:** I Universidade de São Paulo. São Paulo, São Paulo, Brazil; II Prefeitura do Município de São Paulo. São Paulo, São Paulo, Brazil

**Keywords:** Patient Safety, Emergency Medical Services, Patient Care Team, Emergency Mobile Units, Nurses, Seguridad del Paciente, Servicios Médicos de Urgencia, Grupo de Atención al Paciente, Unidades Móviles de Emergencia, Enfermeras y Enfermeros

## Abstract

**Objectives:**

to understand nurses’ experience regarding patient safety in mobile pre-hospital care.

**Method:**

a qualitative, exploratory and descriptive study, conducted with nurses active in mobile pre-hospital care services. Semi-structured interviews were conducted, audio-graved and submitted to Bardin’s content analysis.

**Results:**

from four thematic categories established, nurses reported the care and management skills necessary to work in this service. They demonstrated a commitment to ensuring safe care for patients, staff and spectators. They highlighted the actions taken to prevent and mitigate incidents. However, they based their experiences on practice protocols and individual actions, expressing the need to improve knowledge about patient safety.

**Final Considerations:**

mobile pre-hospital care nurses’ experience in relation to patient safety was limited, suggesting the need for training on the subject, alignment of work processes and implementation of strategies, aiming to guarantee safe care.

## RESUMEN

**Objetivos:**

comprender la experiencia de enfermeros en relación con la seguridad del paciente en la atención prehospitalaria móvil.

**Métodos:**

estudio cualitativo, exploratorio y descriptivo, realizado con enfermeros actuantes en servicios de atención prehospitalaria móvil. Se realizaron entrevistas semiestructuradas, audiogravadas y sometidas al análisis de contenido de Bardin.

**Resultados:**

a partir de cuatro categorías temáticas establecidas, los enfermeros informaron las habilidades de atención y gestión necesarias para actuar en este servicio. Demostraron un compromiso para garantizar una atención segura para los pacientes, el personal y los espectadores. Destacaron las acciones tomadas para prevenir y mitigar incidentes. Sin embargo, basaron sus experiencias en protocolos de práctica y acciones individuales, expresando la necesidad de mejorar el conocimiento sobre la seguridad del paciente.

**Consideraciones Finales:**

la experiencia de los enfermeros que actúan en la atención prehospitalaria móvil en relación a la seguridad del paciente ha sido limitada, sugiriendo la necesidad de capacitación en el tema, alineación de procesos de trabajo e implementación de estrategias, con el objetivo de garantizar una atención segura.

## INTRODUCTION

Admittedly, the last few decades have been marked by advances in healthcare service organization and structuring, with the aim of providing assistance that responds to individuals’ needs and expectations and that produces effective and safe results, regardless of the scope of care^(1)^.

In this context, mobile pre-hospital care services (APHM - *atendimento pré-hospitalar móvel*) stand out, responsible for “early assistance outside hospital settings, provided to individuals in urgent and emergency situations, at risk of intense suffering, sequelae or death and at transport appropriately to a healthcare service”^(2)^. These health equipment have received great prestige for the unique and relevant work developed in several countries^(3)^, including Brazil, with a reduction in mortality rates, especially those arising from external causes^(4,5)^.

In Brazil, in 2003, the Mobile Emergency Care Service (SAMU - *Serviço de Atendimento Móvel de Urgência*) was implemented as part of the government strategy to reorganize emergency care and insert APHM into the public health system^(6)^. Since then, there has been a considerable expansion of this resource throughout the national territory, reaching 85% of citizens in 67.3% (3,750) of municipalities in 2019^(7)^.

The work process at SAMU is understood in two dimensions, such as care and management. In the first dimension, care begins with the Emergency Regulation Center (CRU - *Central de Regulação das Urgências*) team and continues with basic life support (BLS) and/or advanced life support (ALS) teams, which are sent to provide APHM^(2,6)^. Within the scope of the managerial dimension, action planning, team management, logistical provision, integration with other components of the Emergency Care Network (RAU - *Rede de Atenção às Urgências*), monitoring of quality indicators, team training and service assessment^(6)^. Professional nurses actively participate in both dimensions.

It is observed that work environments at SAMU are unstable, the care provided by the teams is complex and heterogeneous^(8)^, and can result in patient harm^(9)^. In this context, there must be a continuous concern for patient safety, which is understood as a structure of organized activities that creates cultures, processes, procedures, behaviors, technologies and environments in healthcare, with the purpose of making errors less likely and reducing risks in a consistent and sustainable way^(10)^.

In international literature, there is a fruitful concern with safety in APHM, focusing mainly on the occurrence of adverse events and near-accidents, occupational risks, safety of decisions not to transport patients and incident prevention from the perspective of organizations and everyone involved^(11-14)^.

Despite the Brazilian National Patient Safety Program (PNSP - *Programa Nacional de Segurança do Paciente*) creation, which aims to contribute to healthcare qualification in all health establishments^(15,16)^, little is known about how actions are managed and developed in APHM services, given the non-obligatory nature of a Patient Safety Center (PSC)^(16)^. It is worth noting that, in primary^(17)^ and hospital^(18)^ care, overwhelming results of incidents are evident; however, there are advances in structuring activities to improve patient safety.

It is worth mentioning the leading role that nurses play in healthcare institutions. Meanwhile, front-line workers, who provide direct patient care, representing the largest contingent among healthcare professionals, have the necessary skills to provide safe care. Authors highlight that these professionals are facilitators in work processes, have the ability to work as a team, communicate effectively, manage risks, recognize the factors that influence patient safety and respond to adverse events^(19,20)^.

Investigations conducted by Brazilian nurses synthesized strategies to ensure safety in APHM and pointed to effective written communication, professional training and the availability of human and material resources as key elements for patient safety^(21)^. Another study proposed steps for patient safety in APHM, which are: patient identification and severity using a wristband, especially in cases of incidents with multiple victims; actions to prevent infection; safe medication administration; security and standardization in the packaging of equipment and materials; attention to service specificities; patient and family engagement when possible; effective communication with the regulatory center; fall, trauma and skin injury prevention; and sustainable use of resources available in the ambulance^(22)^. However, it is important to highlight that national research on the topic is still scarce.

In this way, understanding nurses’ experience of patient safety in this setting can reveal elements of professional practice and support service management in promoting changes in the practice environment. In view of these arguments, it is considered that expanding discussions about this construct in APHM is essential, and this study asks: what is nurses’ experience regarding patient safety in APHM?

## OBJECTIVES

To understand nurses’ experience regarding patient safety in APHM.

## METHODS

### Ethical aspects

The research was initiated after analysis and approval by the Research Ethics Committee (REC) of the *Universidade de São Paulo* School of Nursing, and developed in accordance with Resolution 466/2012^(23)^. When inviting, the researchers clarified the research objective, anonymity guarantee, voluntariness, the risks and benefits of participation and the signing of the Informed Consent Form (ICF) in two copies. To guarantee anonymity, participants’ names were suppressed and coded with the letter E, followed by corresponding cardinal numerals.

### Study design

This is a study with a qualitative, descriptive and exploratory approach, as it seeks to understand phenomena within their context of intervention, establishing links between concepts, representations, beliefs and behaviors, respecting intersubjectivity^(24)^. To guide the dissemination of the results of this investigation, the COnsolidated criteria for REporting Qualitative research (COREQ) criteria were followed^(25)^.

### Theoretical-methodological framework

The term “experience”, used historically by Martin Heidegger, concerns what human beings learn from the place they occupy in the world and the actions they perform. Through it, individuals understand themselves and their meaning in the world of life. It is from this ontology that they open themselves up to understanding others and the world. Experience feeds reflection and is expressed in language. However, experience presentation through language does not bring pure experience, as facts come interpreted, where what is narrated and what is experienced are embedded in and by culture, preceding the narrative and the narrator^(26)^.

### Study participants

Nurses who worked in APHM services for at least one year participated, understanding that the experience is premised on the lived fact^(25)^. Thus, this temporality was established to understand this phenomenon.

To recruit participants, non-probabilistic “snowball” sampling technique was used, whose people selected for the study indicate and/or invite new people from their relationship network^(27)^, with the purpose of obtaining a greater plurality of professional profiles and performance in the different healthcare services that provide APHM.

The execution of the technique began with the nomination of a “seed” participant from the contact network of members of a research group at a public university and, later, through the nomination among successively invited participants, based on their own professional network and that met the criteria. This way, the sampling frame can grow with each interview.

The invitation to participate was sent electronically (e-mail and WhatsApp^®^). For those who indicated interest in sharing their experiences, an in-person meeting was scheduled to obtain consent and carry out the interview on a date, time and place (with privacy, comfort and little noise) chosen by the participants themselves.

To close the sample, the theoretical data saturation criterion was used, i.e., when speeches present repetition of information and there are no new elements for analysis, this represents a criterion for sample sufficiency in qualitative research^(28)^.

### Study setting

The nurses participating in this study were predominantly working in the SAMU in the Metropolitan Region of São Paulo, one of the most populous in the world and the largest in Brazil, with approximately 21 million inhabitants.

The SAMU, in this investigation setting, were distributed in 26 municipalities, had 14 CRU, 358 mobile emergency units^(29)^ and did not have an NSP. It is noteworthy that the actions carried out by the Continuing Education Center (CEC) were not investigated, considering the data collection method characteristics and the diversity of settings.

### Data collection and organization

Data were collected between April and July 2022. The interviews were carried out individually, mediated by authors 1 and 4 of this article, who have previous experience in the type of methodological approach and in APHM.

A semi-structured questionnaire was applied containing information regarding professional sociodemographic and work characteristics and their experience regarding patient safety in APHM, namely: what is your experience regarding patient safety in your APHM? What is the relationship between team safety and patient safety at APHM? What actions and measures are used in your APHM service to promote patient safety? What is the role of nurses in patient safety at APHM?

Each interview was carried out in just one meeting, recorded only in audio, preserving participants’ image, and lasted an average of 20 minutes.

### Data analysis

To systematize the narratives, the transcription and textualization phases were used^(30)^. The interviews were transcribed in full in Microsoft Word^®^ by the same researchers who collected the data, double-checking the transcribed content. Then, textualization was carried out, in order to make the narrative more understandable for readers. Textualizations were read and re-read in search of guiding principles for coding and categorizing information, according to study objective.

After this process, all the material was subjected to content analysis of Bardin’s framework, in the thematic modality, which must be conducted in three stages: 1) pre-analysis (material organization); 2) material exploration (in-depth study of material, using hypotheses and theoretical frameworks); and 3) inferential analysis (step in which the material must be treated through coding, classification and interpretation)^(31)^.

## RESULTS

Seven nurses and seven nurses participated in the study, totaling 14 professionals, with an average age of 43 years (SD=7.19). The mean time working in APHM services was 12 years (SD=5.52).

Professionals’ narratives culminated in the formation of four thematic categories, namely: The role of nurses in mobile pre-hospital care; Patient and team safety in mobile pre-hospital care; Systematization of practices in mobile pre-hospital care and measures related to patient safety; Gaps related to patient safety in mobile pre-hospital care.

### The role of nurses in mobile pre-hospital care

Participants reported the care and management skills necessary to work in APHM services. The main work activities carried out were team and quality management, following care protocols, leadership, training workers and ensuring no harm to patients. These aspects are revealed in the following statements:

*Guide the team in their service context so that at no time do we violate what is already our protocol.* (E2)

*The role of nurses is large and important, as we act as team leaders. Everything that happens inside the ambulance involves greater responsibility for the nurses, so they have to pay attention to everything.* (E6)

*Most of the time, depending on the case, nurses have deeper and more specialized training, and with their experience focused on training, they can collaborate to guarantee better pre-hospital care for patients, seeking to stabilize and improve the clinical picture and not cause an act of iatrogenic.* (E9)

### Patient and team safety in mobile pre-hospital care

In this category, participants’ reports demonstrated commitment to ensuring safe care for everyone involved, patients, workers and spectators. It is observed that providing care without causing harm, safeguarding the physical and mental integrity of patients and promoting quality care, is an important prerogative for these professionals, who at certain times juxtapose their own safety and that of the team.

*Before we enter the victim’s home, we must check the scene, context and the situation involved. It is a differentiated service in which we go to the victim, and not the victim who comes to us. This uncertain environment does not bring much security to the team, as we work in the “dark”.* (E4)

*I think we preserve patient safety, even more than the team itself*. (E5)

*Personally, I always try to prioritize that the patient does not worsen beyond what is already happening to them at that moment. Everyone on the team has this concern.* (E6)

*We work with the maxim of “safe scene”. If the scene is safe, care will be safe for staff and patients alike. However, I have had a situation in which patients fell off the stretcher. It turned over, so the risk of trauma is very high.* (E11)

*When driving an ambulance stretcher, any failure to be careful can cause the wheel to lock and tip to one side, causing harm to patients.* (E14)

The work environment at APHM is multifaceted, unpredictable and imposes constant challenges on professionals. Concern and the need for constant scene assessment can be seen in speeches, which is the first action in care chain, essential for team safety and, consequently, patient safety, preventing the occurrence of new health problems.

*Protecting team safety, we are ensuring that professionals are able to care for patients. Firstly, we have to guarantee our safety and then offer adequate care to victims.* (E4)

*Our safety comes first. If we get hurt, we won’t be able to treat patients, so, assuring myself, I will also be protecting this citizen’s care.* (E7)

*First of all, always our safety. If there is a patient at any type of risk and the team too, we are advised to protect ourselves, because we cannot become a new victim and, mainly, because we are the only team there. If one of us suffers harm or something, who will provide care for patients?* (E1)

[...] *ensuring team safety, we will avoid having another victim on the scene. In this case, victims would be us at SAMU: the nurse, the driver or an assistant and a nursing technician* [...]. (E10)

*We have in mind that we cannot become a new victim and make the patient’s situation worse. They already have a demand. They already have a problem and we cannot increase this problem.* (E3)

[...] *it is understood that, for patients to be safe during pre-hospital care, the team first has to be safe and promote safety at the scene.* (E13)

*First, if I’m not safe to perform the service, I won’t be able to do it properly, so the scene has to be protected so I can apply everything we train, what we know how to do and what patients need.* (E12)

### Systematization of practices in mobile pre-hospital care and measures related to patient safety

In this category, the performance of service management is observed regarding the implementation of systematized practices that contribute to minimizing risks in APHM. In the interviews, it is clear that nurses’ experiences are guided by institutional guidelines with continuous training for their implementation, where each member and the team as a whole follow the same protocols and become co-responsible for care.

*In reality, our institution has recommended a protocol that aims not only to ensure the conditions of the victims and also of the team.* (E2)

*We constantly undergo procedural training according to their needs to review different protocols already established.* (E3)

[...] *if we direct patients to the right entry point, we prioritize and provide adequate care, so we can meet safety goals again. This patient will have a faster response time and less compromise to their health.* (E11)

*From the moment the team arrives to provide care to victims, we will use the best technique necessary to assist them, always valuing safety links* [...]. (E9)

Participants realize that the care provided can cause risks and harm to patients and report the actions taken, aiming to prevent and mitigate incidents and health problems. The proximity to the international goals proposed by the World Health Organization (WHO) and the Brazilian National Health Regulatory Agency (ANVISA - *Agência Nacional de Vigilância Sanitária*) is noted.

### Gaps related to patient safety in mobile pre-hospital care

Despite receiving training and qualifications to exercise institutional protocols and care in APHM, it appears that some nurses expressed limited and fragile experiences related to the specific topic of patient safety, as evidenced by:

**Chart 1:** Measures associated with international patient safety goals and actions carried out by nurses working in mobile pre-hospital care, São Paulo, São Paulo, Brazil, 2022

Patient safety measures	Actions performed by nurses in mobile pre-hospital care
Correct patient identification	*In general, we first confirm patient identification by asking their name and checking documentation. In other situations, when the victim is unknown and does not have personal data, we identify them by describing their age, sex and clothing characteristics.* (E7) *Regarding patient identification, we have two types of patients: conscious and unconscious. We always ask the conscious person their name before carrying out any procedure.* (E8) *The first thing we do with patient safety and goals in mind is to try to identify who the victim is.* (E13)
Clear and assertive communication between professionals and healthcare services	*We always exchange information with the doctor as a safety measure.* (E8) *The regulation center reports an incident, but we will only really know what is happening when we arrive at the scene. This situation causes a certain insecurity and difficulty because we don’t know what we are going to find.* (E4) *At the hospital, really passing on case information effectively. Try to ensure that all data that was collected at the scene reaches the destination location.* (E12)
Injury and fall prevention	*If you need to puncture patients, we normally try to do it assertively. If, on the first attempt, you are unable to do it, do not keep trying several times, to avoid other injuries related to the venipuncture technique.* (E11) *We advise on the correct way to handle the ambulance stretcher, “lowering it to the floor”, as it poses a risk to patients.* (E6) *When transferring patients to another service, it is noted that they spend a long time on our stretchers due to lack of beds, and this has caused falls. Now we are leaving the stretchers lowered to the floor. I think we have to pass this information on to nursing assistants and technicians at our workplace too, so they can be careful so patients don’t have the risk of fall*ing. (E10)
Safe medication administration	*Regarding medication, for instance, we always check medication validity and some equipment that also have a pre-determined maintenance date at the beginning of the shift.* (E6) *We confirm if you have a drug allergy and underlying illnesses.* (E7) *If the doctor tells us to take medication, we first confirm the prescription* [drug, route, dosage] *and then double-check it before administering it.* (E8)
Infection control	*Every procedure begins with the use of personal protective equipment. We have to use PPE to preserve our safety*. (E4) *If you puncture a venous access and do not fix it correctly, the catheter will probably come out, so you will expose patients to a new invasive and risky procedure.* (E14)

*I have worked at APH since 2004 and have little experience regarding patient safety. We don’t have protocol here at* [workplace] *for this.* (E8)

*My experience regarding pre-hospital patient safety is actually very succinct. There are not many studies on this and we are not as focused on practice.* (E1)

*My experience is very fragile in terms of patient safety, because pre-hospital care doesn’t allow us to have things under much control.* [...]. (E12)

## DISCUSSION

Regarding participant sociodemographic characteristics, there was an average age of 43 years and an equal number of male and female nurses. This characteristic can be attributed to the sample composition method of this research. Different results were found in studies in Brazil, indicating that the majority of nursing professionals were female and aged between 35 and 45 years old^(32,33)^. Internationally, in APHM services, in Sweden and Portugal, they indicated a prevalence of men aged between 37 and 43 years, in that order^(34,35)^.

Nurses’ mean working time in APHM was 12 years (SD=5.52), differentiating from other studies carried out in the states of Goiás and São Paulo, whose average varied between 4.7 and 7.9 years, respectively^(8,36)^. Previous investigations carried out with nurses working at APHM, in Sweden and Norway, showed that longer working time in this service was positively associated with greater professional competence^(34,37)^.

In relation to the role of APHM nurses, the results highlight the multiple skills they play in this area of work with teams. In this context, it appears that these professionals’ work involves the need for technical-scientific knowledge, skills, abilities and other responsibilities which are not restricted only to direct patient care, but also to managerial functions related to service organization, leadership, input provision, training and team management^(38,39)^.

Concerning patient safety in APHM, these professionals’ concern and commitment to provide adequate care were noted. In this aspect, authors highlight the role of nurses in guaranteeing and implementing this aspect, reducing avoidable damages associated with care in this context^(40)^.

An investigation conducted in a SAMU, in Brazil, with the objective of analyzing the occurrence of safety incidents during APHM, revealed non-compliance in the assistance provided and circumstances with the potential to cause patient harm related to the goals of patient identification, effective communication, medication safety, risk of infection, prevention of falls and pressure injuries^(41)^.

Nurses have the ability to detect and correct in advance many of the failures that occur in provision of care, especially those capable of compromising patient safety^(38)^. However, it is worth noting that safety is an essential dimension resulting from the responsibility and participation of all team members, service management^(10)^ and, when possible, in the context of APHM, patients and family members.

It is observed that some factors most commonly contribute to the occurrence of safety-related incidents in APHM. A retrospective study, carried out in Sweden, identified that, of the 1,080 medical records evaluated, in 46 (4.3%) adverse events occurred, the main reasons being breaches of the care protocol and incomplete documentation^(11)^. Another prospective survey, carried out in Spain, identified 194 notifications related to patient safety, of which 112 resulted in incidents; of these, 89.7% were observed after assistance and 10.3% before arriving at patient care location^(42)^. In Qatar, a study showed that, of the 3,475 occurrences observed, 161 presented adverse events related to medications, with a predominance of failures in supplying and administering the wrong dosage and outside the recommended dosage^(43)^.

In this regard, the importance of knowledge regarding patient safety, action planning, alignment of work processes, team training and notifications for management and service improvement is evident.

The detection, notification and monitoring of adverse events is a key component for the development of strategies aimed at reducing them. However, with regard to APHM in Brazil, it is clear that this culture was consolidated, evidenced by the lack of records of this information. The annual report on incidents related to healthcare in Brazil, between July 2022 and June 2023^(44)^, does not present data directly related to APHM services.

Considering the complexity of assistance in this type of care, the commitment of national regulatory agencies and competent bodies to implement regulations that guide health safety actions in this environment becomes paramount and emerging^(41)^.

Concerning team safety, it was verified, through nurses’ speeches, constant care in ensuring safe conditions for all professionals, protecting them from any physical and/or emotional harm they may have in the face of APHM. In this line of thought, the literature emphasizes that mobile unit teams must, firstly, ensure that the place of care is safe, that care is carried out efficiently and with the least possible exposure^(45)^.

A study with the objective of understanding the difficulties encountered by the nursing team during APHM, in a SAMU, in the state of Bahia, identified risk situations related to the occurrence of traffic accidents with the ambulance, occupational violence, exposure of images of professionals through photographic records and interpersonal conflict due to the population’s lack of understanding regarding this service’s work process^(46)^.

As for systematization of care practices in APHM, it was observed that nurses are instructed by organizational guidelines with qualifications and training to carry out their tasks, without explaining the safety dimension or offering specific training on the topic. However, they realize that assistance can cause harm to patients, and thus, they carry out actions aimed at preventing and mitigating incidents.

The absence of systemic and organizational actions specifically aimed at the safety of care results in individual actions by each professional, within their practical area, based on their own knowledge.

Research carried out in a SAMU in Brazil revealed that at APHM patients treated in this service were not identified (100%); the records of information in medical records were incomplete (98.3%); medications were prepared inappropriately (61.8%); professionals did not perform hand hygiene (92.8%) or change gloves between procedures (69.1%); and did not check direct contact between patients’ skin and the equipment (72.6%)^(41)^.

Authors highlighted the negative impact of adverse events on professionals in APHM services in Germany. The findings of this investigation showed that, of the 401 doctors interviewed, 53.1% experienced some suffering due to the occurrence of an adverse event to patients; 48.8% had the support of their colleagues to cope; and 11.3% had not fully recovered from this situation^(47)^.

Some measures contribute to better safety within the scope of APHM, including: operationality and configuration of ambulances; proper operation and handling of equipment and stretcher; culture and comprehensive policy in medication administration; structured transfer of care; communication and psychomotor skills to carry out high-risk procedures; conditioning and physical and mental health of professionals; fatigue management; and conducting interprofessional education^(48)^.

A study carried out in APHM services in Spain revealed a positive experience regarding safety from patients’ and families’ perspective, and the main reasons were related to quick access to the service, qualified assistance provided by the interprofessional team and focused on their needs, clear information regarding their health condition and equipment availability to provide care. At the same time, professionals expressed feeling unsafe when working in an unfamiliar environment, without privacy and in ambulances with limited space to carry out their activities^(49)^.

Nurses’ experience regarding patient safety in this research is based on individualized practice protocols and actions. It was observed that professionals showed concern and carried out interventions with the purpose of reducing risks of care, but reported the need to expand knowledge on the topic.

Research carried out in ambulance services in England demonstrated that professionals had varied perceptions about patient safety, that the culture of reporting incidents was weak and the main factors were the lack of a system for reporting and fear of punishment^(50)^.

Research into ambulance services in Sweden, aiming to explore nurses’ experiences and behaviors in near miss situations relating to patient safety, identified that the reasons related to critical incidents are the lack of technical-scientific knowledge, lack of workers’ experience and increased response time in assistance. Furthermore, they indicated fear in reporting the occurrence of injuries to their leaders^(51)^.

In view of these findings, there is a need for integrated organizational approaches that favor the commitment of everyone involved, with safe practice implementation, risk reduction and notification of adverse event occurrence.

### Study limitations

The limitations of this study lie in the scarcity of research with this focus, in the context of APHM services, in the national territory, which makes comparisons impossible and restricts the discussion of findings and convenience sampling, which although it was a representative number, making it possible to reach data saturation, cannot generalize the results.

### Contributions to nursing, health or public policy

Considering the above, the innovative nature of this research stands out in nurses’ experience regarding patient safety in APHM. The findings make significant contributions and point to the need for investments that can support APHM service management, namely: (1) due to the complexity, specificity and relevance of this type of care, work processes must be aligned, enabling the guarantee of systematic, effective and safe care; (2) develop actions that raise awareness, engage and incorporate the idea of collective and shared responsibility in this regard; (3) carry out specific training on the topic so that it is possible to obtain better results in terms of professionals’ experience and the assistance provided; (4) implement strategies aimed at reporting adverse events. Furthermore, this study can encourage further investigations into patient safety in the context of APHM in different settings.

## FINAL CONSIDERATIONS

The work environments at APHM are unstable, and the activities carried out by teams can result in risks to patient safety. Nurses reported the care and management skills necessary to work in APHM services, emphasizing the commitment to providing adequate and safe care to everyone, including patients, professionals and bystanders.

It was found that nurses’ experiences regarding patient safety were fragile and limited and, above all, were mainly based on practice protocols and individual actions. Professionals expressed the need to improve knowledge on the subject.

That said, it is noted that such findings constitute opportunities for improvement in APHM services, aiming to encourage the collective construction of commitments to patient safety, train professionals with the purpose of reducing risks, qualifying care and promoting changes in the practice environment.
