# Supplementary material for: Nurses’ experience regarding patient safety in mobile pre-hospital care
Source: Rev Bras Enferm. 2024 Dec 13;77(5):e20230529. doi: 10.1590/0034-7167-2023-0529 (PMC11654230; doi:10.1590/0034-7167-2023-0529)
Supplement: Translation

# Translation: Experiência de enfermeiros relativa à segurança do paciente no atendimento pré-hospitalar móvel

**DOI:** 10.1590/0034-7167-2023-0529pt

**Authors:** Eduardo Dias Filipe, Roberto Chrispim Modesto, Hercules de Oliveira Carmo, Haviley Oliveira Martins, Maristela Santini Martins

**Affiliations:** I Universidade de São Paulo. São Paulo, São Paulo, Brasil; II Prefeitura do Município de São Paulo. São Paulo, São Paulo, Brasil

**Keywords:** Segurança do Paciente, Atendimento Pré-Hospitalar, Equipes de Assistência ao Paciente, Unidades Móveis de Emergência, Enfermeiras e Enfermeiros

## Abstract

**Objetivos:**

compreender a experiência de enfermeiros relativa à segurança do paciente no atendimento pré-hospitalar móvel.

**Métodos:**

estudo qualitativo, exploratório e descritivo, realizado com enfermeiros atuantes em serviços de atendimento pré-hospitalar móvel. Realizaram-se entrevistas semiestruturadas, audiogravadas, submetidas a análise de conteúdo de Bardin.

**Resultados:**

a partir de quatro categorias temáticas estabelecidas, enfermeiros relataram as competências assistenciais e gerenciais necessárias para atuação neste serviço. Demonstraram compromisso em garantir um cuidado seguro para pacientes, equipes e espectadores. Evidenciaram as ações realizadas para prevenção e mitigação de incidentes. Contudo, pautaram suas experiências em protocolos de práticas e ações individuais, expressando a necessidade de aprimorar o conhecimento sobre a segurança do paciente.

**Considerações Finais:**

a experiência dos enfermeiros atuantes no atendimento pré-hospitalar móvel em relação à segurança do paciente foi limitada, sugerindo a necessidade de capacitação sobre a temática, alinhamento dos processos de trabalho e implementação de estratégias, visando à garantia de cuidados seguros.

## INTRODUÇÃO

Reconhecidamente, as últimas décadas foram marcadas por avanços na organização e estruturação dos serviços de saúde, com o objetivo de proporcionar uma assistência que responda às necessidades e expectativas dos indivíduos e que produza resultados eficazes e seguros, independentemente do âmbito de atenção^(1)^.

Nesse contexto, destacam-se os serviços de atendimento pré-hospitalar móvel (APHM), responsáveis pela “assistência precoce e fora do ambiente hospitalar, realizada a indivíduos em situações de urgência e emergência, com risco de sofrimento intenso, sequela ou morte e ao transporte de maneira adequada a um serviço de saúde”^(2)^. Esses equipamentos de saúde têm recebido grande prestígio pelo trabalho singular e relevante desenvolvido em diversos países^(3)^, inclusive no Brasil, com diminuição das taxas de mortalidade, principalmente aquelas advindas de causas externas^(4,5)^.

No Brasil, no ano de 2003, houve a implantação do Serviço de Atendimento Móvel de Urgência (SAMU) como parte da estratégia governamental na reorganização da atenção às urgências e inserção do APHM no sistema público de saúde^(6)^. Desde então, nota-se considerável expansão desse recurso em todo o território nacional, alcançando 85% dos cidadãos em 67,3% (3.750) dos municípios em 2019^(7)^.

O processo de trabalho no SAMU é compreendido em duas dimensões, como assistencial e gerencial. Na primeira dimensão, o atendimento começa com a equipe da Central de Regulação das Urgências (CRU) e continua com as equipes de suporte básico de vida (SBV) e/ou suporte avançado de vida (SAV), que são enviadas para prestar o APHM^(2,6)^. No âmbito da dimensão gerencial, ocorrem o planejamento das ações, a gestão das equipes, o provimento logístico, a integração com os outros componentes da Rede de Atenção às Urgências (RAU), o monitoramento dos indicadores de qualidade, a capacitação das equipes e a avaliação dos serviços^(6)^. O profissional enfermeiro participa ativamente de ambas as dimensões.

Observa-se que os ambientes de trabalho no SAMU são instáveis, os atendimentos realizados pelas equipes são complexos e heterogêneos^(8)^, e podem resultar em danos ao paciente^(9)^. Nesse contexto, deve haver uma preocupação contínua com a segurança do paciente, que é entendida como uma estrutura de atividades organizadas que cria culturas, processos, procedimentos, comportamentos, tecnologias e ambientes nos cuidados de saúde, com o propósito de tornar os erros menos prováveis e de reduzir os riscos de forma consistente e sustentável^(10)^.

Na literatura internacional, nota-se profícua preocupação com a segurança no APHM, concentrando-se principalmente na ocorrência dos eventos adversos e dos quase acidentes, nos riscos ocupacionais, na segurança das decisões de não transporte dos pacientes e na prevenção de incidentes sob a perspectiva das organizações e de todos os envolvidos^(11-14)^.

No Brasil, apesar da criação do Programa Nacional de Segurança do Paciente (PNSP), que visa contribuir para a qualificação do cuidado em saúde em todos os estabelecimentos de saúde^(15,16)^, pouco se sabe sobre como são gerenciadas e desenvolvidas as ações nos serviços de APHM, visto a não obrigatoriedade de um Núcleo de Segurança do Paciente (NSP)^(16)^. Cabe pontuar que, na atenção primária^(17)^ e hospitalar^(18)^, evidenciam-se resultados avassaladores de incidentes; todavia, verificam-se avanços no que tange à estruturação das atividades para melhoria da segurança do paciente.

É oportuno mencionar o protagonismo que os enfermeiros têm nas instituições de saúde. Enquanto isso, trabalhadores da linha de frente, que prestam cuidados diretos ao paciente, representando o maior contingente entre profissionais de saúde, possuem as competências necessárias para a prestação de uma assistência segura. Autores destacam que esses profissionais são facilitadores nos processos de trabalho, têm capacidade para trabalhar em equipe, comunicar de forma eficaz, gerir riscos, reconhecer os fatores que influenciam a segurança do paciente e responder aos eventos adversos^(19,20)^.

Investigações conduzidas por enfermeiros brasileiros sintetizaram estratégias para garantir a segurança no APHM e apontaram a comunicação escrita efetiva, o treinamento dos profissionais e a disponibilidade de recursos humanos e materiais como elementos-chave para a segurança do paciente^(21)^. Outro estudo propôs passos para a segurança do paciente no APHM, que são: identificação do paciente e da gravidade por meio de pulseira, principalmente nos casos de incidentes com múltiplas vítimas; ações para a prevenção de infecção; administração segura de medicamentos; segurança e padronização no acondicionamento de equipamentos e materiais; atenção para as especificidades do serviço; engajamento do paciente e da família, quando possível; comunicação efetiva com a central de regulação; prevenção de quedas, traumas e lesões de pele; e utilização sustentável dos recursos disponíveis na ambulância^(22)^. No entanto, é importante salientar que pesquisas nacionais sobre o tema ainda são escassas.

Desse modo, a compreensão da experiência dos enfermeiros sobre a segurança do paciente nesse cenário de atuação pode revelar elementos da prática profissional e subsidiar a gestão dos serviços na promoção de mudanças no ambiente de prática. Tendo em vista tais argumentos, considera-se que a ampliação de discussões acerca desse construto no APHM é essencial, e este estudo pergunta: qual a experiência dos enfermeiros relativa à segurança do paciente no APHM?

## OBJETIVOS

Compreender a experiência de enfermeiros relativa à segurança do paciente no APHM.

## MÉTODOS

### Aspectos éticos

A pesquisa foi iniciada após análise e aprovação pelo Comitê de Ética em Pesquisa (CEP) da Escola de Enfermagem da Universidade de São Paulo, e desenvolvida em conformidade com a Resolução nº 466/2012^(23)^. No ato do convite, os pesquisadores esclareceram sobre o objetivo da pesquisa, a garantia do anonimato, a voluntariedade, os riscos e benefícios da participação e a assinatura do Termo de Consentimento Livre e Esclarecido (TCLE) em duas vias. Para garantia do anonimato, os nomes dos participantes foram suprimidos e codificados com a letra E, seguida de numerais cardinais correspondentes.

### Tipo de estudo

Trata-se de estudo de abordagem qualitativa, descritiva e exploratória, pois busca a compreensão de fenômenos dentro do seu contexto de intervenção, estabelecendo ligações entre conceitos, representações, crenças e comportamentos, respeitando a intersubjetividade^(24)^. Para guiar e nortear a divulgação dos resultados desta investigação, seguiram-se os critérios do *COnsolidated criteria for REporting Qualitative research* (COREQ)^(25)^.

### Referencial teórico-metodológico

O termo “experiência”, utilizado historicamente por Martin Heidegger, diz respeito ao que o ser humano apreende no lugar que ocupa no mundo e nas ações que realiza. Através dela, o indivíduo compreende a si mesmo e ao seu significado no mundo da vida. É a partir dessa ontologia que ele se abre para entender os outros e o mundo. A experiência alimenta a reflexão e se expressa na linguagem. Entretanto, a apresentação da experiência pela linguagem não traz a experiência pura, pois os fatos vêm interpretados, onde o narrado e o vivido por si estão entranhados na e pela cultura, precedendo à narrativa e ao narrador^(26)^.

### Participantes do estudo

Participaram enfermeiros(as) que atuavam em serviços de APHM há no mínimo um ano, compreendendo que a experiência tem como premissa o fato vivido^(25)^. Assim, estabeleceu-se essa temporalidade para entender esse fenômeno.

Para o recrutamento dos participantes, empregou-se a técnica de amostragem não probabilística “bola de neve”, cujas pessoas selecionadas para o estudo indicam e/ou convidam novas pessoas de sua rede de relacionamento^(27)^, com o propósito de obter maior pluralidade de perfis profissionais e de atuação nos diferentes serviços de saúde que prestam APHM.

A execução da técnica começou a partir da indicação de um participante “semente” da rede de contatos dos membros de um grupo de pesquisa de uma universidade pública e, posteriormente, por meio da nomeação entre participantes sucessivamente convidados, com base na sua própria rede profissional e que contemplasse os critérios. Dessa forma, o quadro de amostragem pode crescer a cada entrevista.

O convite para participar ocorreu por meio eletrônico (*e-mail* e *WhatsApp* ^®^). Àqueles que sinalizaram interesse em compartilhar suas experiências, agendou-se um encontro presencial para obtenção do consentimento e realização da entrevista em data, horário e local (com privacidade, conforto e pouco ruído) escolhidos pelos próprios participantes.

Para o fechamento amostral, utilizou-se o critério de saturação teórica dos dados, ou seja, quando os discursos apresentam repetição de informações e não existem novos elementos para a análise, isso representa critério de suficiência de amostra na pesquisa qualitativa^(28)^.

### Cenário do estudo

Os enfermeiros participantes deste estudo eram, predominantemente, atuantes nos SAMU da Região Metropolitana de São Paulo, uma das mais populosas do mundo e a maior do Brasil, com aproximadamente 21 milhões de habitantes.

Os SAMU, neste cenário de investigação, estavam distribuídos em 26 municípios, tinham 14 CRU, 358 unidades móveis de urgência^(29)^ e não possuíam NSP. Ressalta-se que não foram averiguadas as ações realizadas pelo Núcleo de Educação Permanente (NEP), em vista da característica do modo de coleta de dados e da diversidade de cenários.

### Coleta e organização dos dados

Os dados foram coletados entre os meses de abril e julho de 2022. As entrevistas foram realizadas individualmente, mediadas pelos autores 1 e 4 deste artigo, que acumulam experiências prévias no tipo de abordagem metodológica e no APHM.

Aplicou-se questionário semiestruturado que continha informações relativas à caracterização sociodemográfica e laborais dos profissionais e à experiência sobre a segurança do paciente no APHM, a saber: qual a sua experiência relativa à segurança do paciente nos seus APHM? Qual a relação da segurança da equipe com a segurança do paciente no APHM? Quais ações e medidas são utilizadas no seu serviço de APHM para promover a segurança do paciente? Qual o papel do enfermeiro na segurança do paciente no APHM?

Cada entrevista foi realizada em apenas um encontro, gravadas somente em áudio, preservando a imagem dos participantes, e tiveram duração média de 20 minutos.

### Análise dos dados

Para a sistematização das narrativas, empregaram-se as fases de transcrição e textualização^(30)^. As entrevistas foram transcritas na íntegra no programa *Microsoft Word* ^®^ pelos mesmos pesquisadores que realizaram a coleta dos dados, com dupla checagem do conteúdo transcrito. Em seguida, realizou-se a textualização, a fim de tornar a narrativa mais compreensível para o leitor. As textualizações foram lidas e relidas em busca de eixos norteadores para codificação e categorização das informações, segundo o objetivo do estudo.

Após esse processo, todo o material foi submetido à análise de conteúdo do referencial proposto por Bardin, na modalidade temática, na qual deve ser conduzida em três etapas: 1) pré-análise (organização do material); 2) exploração do material (estudo aprofundado do material, utilizando hipóteses e referenciais teóricos); e 3) análise inferencial (etapa na qual o material deve ser tratado por meio da codificação, classificação e interpretação)^(31)^.

## RESULTADOS

Participaram do estudo sete enfermeiras e sete enfermeiros, totalizando 14 profissionais, com média de idade de 43 anos (DP=7,19). O tempo médio de trabalho em serviços de APHM foi de 12 anos (DP=5,52).

As narrativas dos profissionais culminaram na formação de quatro categorias temáticas, a saber: O papel do enfermeiro no atendimento pré-hospitalar móvel; Segurança do paciente e da equipe no atendimento pré-hospitalar móvel; Sistematização das práticas no atendimento pré-hospitalar móvel e medidas relacionadas à segurança do paciente; Lacunas relativas à segurança do paciente no atendimento pré-hospitalar móvel.

### O papel do enfermeiro no atendimento pré-hospitalar móvel

Os participantes relataram as competências assistenciais e gerenciais necessárias para a atuação nos serviços de APHM. As principais atividades laborais executadas foram gestão das equipes e da qualidade, seguimento dos protocolos assistenciais, liderança, capacitação dos trabalhadores e garantia de não causar danos ao paciente. Esses aspectos são revelados nas seguintes falas:

*Nortear a equipe no seu contexto de atendimento para que em nenhum momento venhamos infringir aquilo que já é nosso protocolo.* (E2)

*O papel do enfermeiro é grande e importante, pois atuamos como líderes da equipe. Tudo que vai acontecer dentro da ambulância tem uma responsabilidade maior para os enfermeiros, então ele tem que estar atento a tudo.* (E6)

*Na maioria das vezes, dependendo do caso, o enfermeiro tem uma formação mais profunda e especializada, podendo com a sua experiência voltada aos treinamentos, colaborar para garantia de uma melhor assistência pré-hospitalar ao paciente, procurando estabilizar e melhorar o quadro clínico e não causar um ato de iatrogenia.* (E9)

### Segurança do paciente e da equipe no atendimento pré-hospitalar móvel

Nessa categoria, os relatos dos participantes demonstraram compromisso em garantir um cuidado seguro para todos os envolvidos, pacientes, trabalhadores e espectadores. Observa-se que prestar um atendimento sem causar danos, salvaguardando a integridade física e mental dos pacientes e promovendo um cuidado de qualidade, é prerrogativa importante para esses profissionais, que em certos momentos justapõem a sua própria segurança e da equipe.

*Antes de nós entrarmos na casa da vítima, devemos verificar a cena, contexto e a situação envolvida. É um atendimento diferenciado em que a gente vai até a vítima, e não ela que vem até nós. Este ambiente incerto não traz tanta segurança para a equipe, pois a gente trabalha no “escuro”.* (E4)

*Eu acho que nós preservamos a segurança do paciente, até mais que da própria equipe.* (E5)

*Eu, particularmente, tento sempre priorizar que o paciente não tenha uma piora além do que já está ocorrendo com ele naquele momento. Todos da equipe têm essa preocupação.* (E6)

*Nós trabalhamos com a máxima de “cena segura”. Se a cena está segura, o atendimento será seguro para a equipe e também para o paciente. Porém, já tive situação de atendimento em que o paciente caiu da maca. Ela virou, então o risco de trauma é muito grande.* (E11)

*Na condução da maca da ambulância, qualquer falha de cuidado, ela pode travar a roda e tombar para o lado, acarretando um dano ao paciente.* (E14)

O ambiente de trabalho no APHM é multifacetado, imprevisível e impõe desafios constantes aos profissionais. Percebem-se nos discursos a preocupação e a necessidade de avaliação constante da cena, sendo essa a primeira ação na cadeia de assistência, elementar para a segurança da equipe e, consequentemente, do paciente, evitando a ocorrência de novos agravos à saúde.

*Resguardando a segurança da equipe, nós estamos garantindo que os profissionais tenham condições de atender o paciente. Em primeiro lugar, temos que garantir a nossa segurança para depois ofertar um atendimento adequado para a vítima.* (E4)

*Nossa segurança vem em primeiro momento. Se nos machucarmos, não vamos atender o paciente, então, eu me assegurando, vou estar também protegendo o atendimento desse cidadão.* (E7)

*Em primeiro lugar, sempre a segurança da gente. Se tem algum paciente correndo qualquer tipo de risco e a equipe também, somos orientados a nos resguardar, porque não podemos virar uma nova vítima e, principalmente, porque somos a única equipe lá. Se algum de nós sofrer algum dano ou alguma coisa, quem vai realizar o atendimento para o paciente?* (E1)

[...] *garantindo a segurança da equipe, vamos evitar ter mais uma vítima na cena. Nesse caso, as vítimas seriam nós do SAMU: o enfermeiro, o condutor ou um auxiliar e um técnico de enfermagem* [...]. (E10)

*Temos na cabeça que não podemos nos tornar uma nova vítima e piorar a situação do paciente. Ele já está com uma demanda, ele já está com problema e não podemos aumentar esse problema.* (E3)

[...] *entende-se que, para o paciente estar seguro no atendimento pré-hospitalar, primeiro a equipe tem que estar segura e promover a segurança da cena.* (E13)

*Primeiro, se eu não estiver segura para realizar o atendimento, não vou conseguir fazer da maneira adequada, então a cena tem que estar protegida para eu conseguir aplicar tudo aquilo que a gente treina, que sabe fazer e que o paciente precisa.* (E12)

### Sistematização das práticas no atendimento pré-hospitalar móvel e medidas relacionadas à segurança do paciente

Nessa categoria, observa-se a atuação da gestão dos serviços quanto à implementação de práticas sistematizadas que concorrem para minimizar os riscos no APHM. Nas entrevistas, evidencia-se que as experiências dos enfermeiros são guiadas por diretrizes institucionais com contínuos treinamentos para a sua execução, onde cada integrante e a equipe como um todo seguem os mesmos protocolos e se tornam corresponsáveis no cuidado.

*Na realidade, nossa instituição tem preconizado um protocolo onde visa não só assegurar as condições da vítima, como também da equipe.* (E2)

*Constantemente, nós passamos por capacitações de procedimentos de acordo com as necessidades que precisam, para revisões de diferentes protocolos já instituídos.* (E3)

[...] *se encaminharmos o paciente para a porta de entrada certa, a gente prioriza e oportuniza um atendimento adequado, então vamos ao encontro das metas de segurança de novo. Esse paciente vai ter um tempo-resposta de atendimento mais rápido e menor comprometimento à sua saúde.* (E11)

*A partir do momento que a equipe chega para prestar os atendimentos a vítima, nós usaremos a melhor técnica necessária para assisti-lo, prezando sempre pelos elos da segurança* [...]. (E9)

Os participantes percebem que a assistência prestada pode acarretar riscos e danos aos pacientes e relatam as ações adotadas, visando à prevenção e mitigação dos incidentes e agravos à saúde. Nota-se a proximidade com as metas internacionais propostas pela Organização Mundial da Saúde (OMS) e pela Agência Nacional de Vigilância Sanitária (ANVISA).

### Lacunas relativas à segurança do paciente no atendimento pré-hospitalar móvel

Apesar de receberem treinamentos e capacitações para o exercício dos protocolos institucionais e do cuidado em APHM, verifica-se que alguns enfermeiros expressaram experiências limitadas e frágeis relativas ao tema específico da segurança do paciente, como evidenciado por:

**Quadro 1:** Medidas associadas às metas internacionais de segurança do paciente e ações executadas pelos enfermeiros atuantes em atendimento pré-hospitalar móvel, São Paulo, São Paulo, Brasil, 2022

Medidas de segurança do paciente	Ações executadas pelos enfermeiros em atendimento pré-hospitalar móvel
Identificação correta de pacientes	*No geral, primeiramente realizamos a confirmação de identificação do paciente perguntando seu nome e verificando a documentação. Em outras situações, quando a vítima é desconhecida e não tem os dados pessoais, nós a identificamos descrevendo a idade, o sexo e as características das vestimentas.* (E7) *Em relação à identificação do paciente, nós temos dois tipos de paciente: os conscientes e os inconscientes. Os conscientes nós sempre perguntamos o nome dele antes de fazer qualquer procedimento.* (E8) *A primeira coisa que nós fazemos pensando na segurança do paciente e nas metas é a tentativa de identificar quem é a vítima*. (E13)
Comunicação clara e assertiva entre profissionais e serviços de saúde	*Sempre realizamos troca de informações com o médico como medida de segurança.* (E8) *A central de regulação passa uma ocorrência, porém só saberemos realmente o que está acontecendo quando chegamos no local. Esta situação causa uma certa insegurança e dificuldade por não sabermos o que vamos encontrar.* (E4) *No hospital, realmente passar as informações do caso de maneira efetiva. Tentar garantir que todos os dados que foram coletados na cena cheguem ao local de desti*no. (E12)
Prevenção de lesões e quedas	*Se precisa puncionar o paciente, normalmente procuramos fazer de forma assertiva. Se, na primeira tentativa, não conseguir fazer, não fica tentando várias vezes, para que se evite outras lesões relacionadas à técnica de punção venosa.* (E11) *Orientamos a forma correta de manipular a maca da ambulância, “abaixando até o chão”, pois ela oferece um risco para o paciente.* (E6) *Na transferência do paciente para outro serviço, nota-se que ele fica muito tempo nas nossas macas por falta de leito, e isso tem ocasionado quedas. Agora estamos deixando as macas abaixadas até o chão. Eu acho que temos que passar essa informação para os auxiliares e técnicos de enfermagem do nosso local de trabalho também, para eles terem cuidado para o paciente não ter o risco de qu*eda. (E10)
Administração segura de medicação	*Em relação à medicação, por exemplo, sempre checamos no início do plantão a validade das mesmas e alguns equipamentos que também têm data de manutenção pré-determinada.* (E6) *Confirmamos se tem alergia medicamentosa e doenças de bases.* (E7) *Se o médico fala para fazer uma medicação, nós confirmamos primeiramente a prescrição* [droga, via, dosagem] *e depois fazemos uma dupla checagem antes de administ*rar. (E8)
Controle de infecção	*Todo procedimento inicia-se com o uso dos equipamentos de proteção individual. A gente tem que utilizar os EPIs para preservar a nossa segurança.* (E4) *Se você punciona um acesso venoso e não faz a fixação correta, provavelmente o cateter irá sair, então você irá expor o paciente a um novo procedimento invasivo e que tem risc*o. (E14)

*Eu trabalho no APH desde 2004 e tenho pouca experiência em relação à segurança do paciente. Não temos protocolo aqui no* [local de trabalho] *para isso.* (E8)

*Minha experiência em relação à segurança do paciente no pré-hospitalar, na verdade, é muito sucinta. Não tem muitos estudos sobre isso e não estamos tão focados na prática.* (E1)

*Minha experiência é bem frágil em questão da segurança do paciente, porque o atendimento pré-hospitalar não deixa a gente ter as coisas muito sob controle* [...]. (E12)

## DISCUSSÃO

Referente às características sociodemográficas dos participantes, constataram-se média de idade de 43 anos e igual número de enfermeiros e enfermeiras. Essa característica pode ser atribuída ao método de composição da amostra desta pesquisa. Resultados diferentes foram encontrados em estudos no Brasil, apontando que grande parte dos profissionais de enfermagem era do sexo feminino e tinha idade entre 35 e 45 anos^(32,33)^. No contexto internacional, em serviços de APHM, na Suécia e em Portugal, apontaram prevalência de homens com idade entre 37 e 43 anos, nessa ordem^(34,35)^.

O tempo médio de trabalho dos enfermeiros no contexto APHM foi de 12 anos (DP=5,52), diferenciando de outros estudos desenvolvidos nos estados de Goiás e de São Paulo, cuja média variou entre 4,7 e 7,9 anos, respectivamente^(8,36)^. Investigações anteriores realizadas com enfermeiros atuantes no APHM, na Suécia e na Noruega, evidenciaram que o maior tempo de trabalho nesse serviço foi positivamente associado a maior competência profissional^(34,37)^.

Em relação ao papel dos enfermeiros no APHM, os resultados ressaltam as múltiplas competências que esses desempenham nesse âmbito de atuação junto às equipes. Nesse contexto, verifica-se que o trabalho desses profissionais envolve a necessidade de conhecimento técnico-científico, aptidões, habilidades e demais responsabilidades que não se restringem somente à assistência direta ao paciente, mas também às funções gerenciais relativas à organização do serviço, à liderança, ao provimento de insumos, aos treinamentos e à gestão das equipes^(38,39)^.

Concernente à segurança do paciente no ambiente do APHM, notaram-se a preocupação e o comprometimento desses profissionais em realizar uma assistência adequada. Nesse aspecto, autores sobrelevam o papel do enfermeiro na garantia e efetivação desse quesito, reduzindo os danos evitáveis associados à assistência nesse contexto^(40)^.

Investigação conduzida em um SAMU, no Brasil, com o objetivo de analisar a ocorrência de incidentes de segurança durante o APHM, evidenciou não conformidade na assistência prestada e circunstâncias com potencial para causar dano ao paciente relacionadas às metas de identificação do paciente, comunicação efetiva, segurança medicamentosa, risco de infecção, prevenção de quedas e lesões por pressão^(41)^.

Os enfermeiros têm a capacidade de detectar e corrigir antecipadamente muitas das falhas que ocorrem na prestação de cuidados, principalmente aquelas capazes de comprometer a segurança dos pacientes^(38)^. Contudo, cabe pontuar que a segurança é uma dimensão essencial resultante da responsabilidade e da participação de todos os membros da equipe, da gestão dos serviços^(10)^ e, quando possível, no contexto do APHM, do paciente e dos familiares.

Observa-se que alguns fatores contribuem mais comumente para a ocorrência de incidentes relacionados à segurança no APHM. Estudo retrospectivo, realizado na Suécia, identificou que, dos 1.080 prontuários avaliados, em 46 (4,3%), ocorreram eventos adversos, tendo como principais motivos a quebra do protocolo de atendimento e a documentação incompleta^(11)^. Outra pesquisa, do tipo prospectiva, realizada na Espanha, identificou 194 notificações relacionadas à segurança do paciente, das quais 112 resultaram em incidentes; desses, 89,7% foram observados após a assistência e 10,3% antes de chegar ao local de atendimento ao paciente^(42)^. No Catar, estudo mostrou que, das 3.475 ocorrências observadas, 161 apresentaram eventos adversos relativos a medicamentos, com predominância em falhas no fornecimento e na administração da dosagem errada e fora do recomendado^(43)^.

Nesse sentido, evidencia-se a importância do conhecimento quanto à temática de segurança do paciente, do planejamento das ações, do alinhamento dos processos de trabalho, da capacitação das equipes e das notificações para gestão e melhoria dos serviços.

A detecção, notificação e monitorização dos eventos adversos é componente-chave para o desenvolvimento de estratégias que visem à sua redução. Entretanto, no que tange ao APHM no Brasil, percebe-se a não consolidação dessa cultura, evidenciada pela ausência de registros dessas informações. O relatório anual sobre os incidentes relacionados à assistência à saúde no Brasil, entre julho de 2022 e junho de 2023^(44)^, não apresenta dados relacionados diretamente aos serviços de APHM.

Considerando a complexidade de assistência nessa modalidade de atenção, torna-se primordial e emergente o empenho das agências reguladoras nacionais e órgãos competentes para a implementação de normativas que orientem as ações de segurança em saúde nesse ambiente^(41)^.

No que tange à segurança da equipe, verificou-se, pelos discursos dos enfermeiros, um cuidado constante em garantir condições seguras para todos os profissionais, resguardando-os de quaisquer danos físicos e/ou emocionais que possam ter perante o APHM. Nessa linha de pensamento, a literatura enfatiza que as equipes das unidades móveis devem, primeiramente, assegurar que o local de atendimento seja seguro, que os cuidados sejam executados de maneira eficiente e com a menor exposição possível^(45)^.

Estudo com o objetivo de compreender as dificuldades encontradas pela equipe de enfermagem durante o APHM, em um SAMU, no estado da Bahia, identificou situações de risco relacionadas à ocorrência de acidentes de trânsito com a ambulância, violência ocupacional, exposição de imagens dos profissionais mediante registros fotográficos e conflito interpessoal pela falta de compreensão da população quanto ao processo de trabalho desse serviço^(46)^.

Relativamente à sistematização das práticas assistenciais no APHM, observou-se que os enfermeiros são instruídos por diretrizes organizacionais com capacitações e treinamentos para a execução das suas tarefas, sem explicitar a dimensão da segurança ou a oferta de capacitações específicas sobre o tema. Porém, percebem que a assistência pode acarretar danos aos pacientes, e assim, realizam ações visando à prevenção e mitigação dos incidentes.

A ausência de ações sistêmicas e organizacionais voltadas especificamente para a segurança da assistência resulta em ações individuais de cada profissional, dentro da sua área prática, a partir do seu próprio conhecimento.

Pesquisa realizada em um SAMU no Brasil revelou que no APHM os pacientes atendidos nesse serviço não eram identificados (100%); os registros das informações nos prontuários estavam incompletos (98,3%); as medicações foram preparadas de maneira inadequada (61,8%); os profissionais não realizavam a higienização das mãos (92,8%) e troca de luvas entre os procedimentos (69,1%); e não verificavam o contato direto da pele do paciente com os equipamentos (72,6%)^(41)^.

Autores destacaram o impacto negativo dos eventos adversos nos profissionais em serviços de APHM na Alemanha. Os achados desta investigação evidenciaram que, dos 401 médicos entrevistados, 53,1% tiveram algum sofrimento em função da ocorrência de um evento adverso ao paciente; 48,8% contaram com o apoio de seus colegas para o enfrentamento; e 11,3% não haviam se recuperado totalmente dessa situação^(47)^.

Algumas medidas concorrem para uma melhor segurança no âmbito do APHM, sendo: a operacionalidade e a configuração das ambulâncias; o funcionamento e o manejo adequado dos equipamentos e da maca; a cultura e a política abrangente na administração de medicamentos; a transferência de cuidados estruturada; as habilidades de comunicação e psicomotoras para execução de procedimentos de alto risco; o condicionamento e a saúde física e mental dos profissionais; o gerenciamento da fadiga; e a realização de educação interprofissional^(48)^.

Estudo realizado em serviços de APHM, na Espanha, revelou uma experiência positiva quanto à segurança na perspectiva dos pacientes e familiares, e os principais motivos estavam relacionados ao rápido acesso ao serviço, à assistência qualificada prestada pela equipe interprofissional e centrada nas suas necessidades, às informações claras quanto à sua condição de saúde e à disponibilidade de equipamentos para a prestação do cuidado. Paralelamente, os profissionais expressaram sentir-se inseguros quando atuavam em ambiente desconhecido, sem privacidade e em ambulâncias com espaço limitado para o desempenho de suas atividades^(49)^.

A experiência dos enfermeiros relativa à segurança do paciente nesta pesquisa está baseada em protocolos de práticas e ações individualizadas. Avistou-se que os profissionais demonstravam preocupação e realizavam intervenções com o propósito de redução dos riscos da assistência, porém relataram a necessidade de ampliar o conhecimento sobre o tema.

Pesquisa realizada em serviços de ambulância, na Inglaterra, demonstrou que os profissionais tinham percepções variadas sobre segurança do paciente, que a cultura de notificação dos incidentes era fraca e os principais fatores foram a falta de um sistema para notificar e o medo de punições^(50)^.

Investigação em serviços de ambulâncias na Suécia, com o objetivo de explorar as experiências de enfermeiros e os comportamentos em situações de quase erros relativos à segurança do paciente, identificou que os motivos relacionados aos incidentes críticos são a falta de conhecimento técnico-científico, a pouca experiência dos trabalhadores e o aumento do tempo-resposta na assistência. Ainda, apontaram medo em reportar as ocorrências dos agravos aos seus líderes^(51)^.

Diante desses achados, nota-se a necessidade de abordagens organizacionais de forma integrada que favoreçam o compromisso de todos os envolvidos, com a implantação de práticas seguras, diminuição dos riscos e notificação da ocorrência dos eventos adversos.

### Limitações do estudo

As limitações deste estudo residem na escassez de pesquisas com este enfoque, no contexto dos serviços de APHM, no território nacional, o que impossibilita comparações e restringe a discussão dos achados e a amostragem por conveniência, que embora tenha sido um número representativo, sendo possível atingir a saturação dos dados, não pode generalizar os resultados.

### Contribuições para a área da enfermagem, saúde ou políticas públicas

Diante do exposto, destaca-se o caráter inovador desta pesquisa ao compreender a experiência de enfermeiros relativa à segurança do paciente no APHM. Os achados trazem contribuições significativas e apontam a necessidade de investimentos que podem subsidiar a gestão dos serviços de APHM, a saber: (1) pela complexidade, especificidade e relevância dessa modalidade de atenção, deve-se alinhar os processos de trabalho, possibilitando a garantia de cuidados sistemáticos, eficazes e seguros; (2) desenvolver ações que sensibilizem, engajem e incorporem a ideia da responsabilidade coletiva e compartilhada nesse quesito; (3) realizar capacitações específicas na temática, para que seja possível obter melhores desfechos quanto à experiência dos profissionais e na assistência prestada; (4) implementar estratégias que visem à notificação dos eventos adversos. Ainda, este estudo pode impulsionar a realização de novas investigações sobre a segurança do paciente no contexto do APHM em cenários diferentes.

## CONSIDERAÇÕES FINAIS

Os ambientes de trabalho no APHM são instáveis, e as atividades executadas pelas equipes podem resultar em riscos à segurança do paciente. Os enfermeiros relataram as competências assistenciais e gerenciais necessárias para a atuação nos serviços de APHM, enfatizando o compromisso de prestar uma assistência adequada e segura a todos, incluindo pacientes, profissionais e espectadores.

Verificou-se que as experiências dos enfermeiros relativas à segurança do paciente eram frágeis e limitadas e, sobretudo, estavam principalmente baseadas em protocolos de práticas e ações individuais. Os profissionais expressaram a necessidade de aprimorar o conhecimento sobre a temática.

Isto posto, pontua-se que tais achados constituem oportunidades de melhoria nos serviços de APHM, visando fomentar a construção coletiva de compromissos com a segurança do paciente, capacitar os profissionais com o propósito de reduzir os riscos, qualificar o cuidado e promover mudanças no ambiente de prática.
